# Wnt pathway antagonists, *SFRP1*, *SFRP2*, *SOX17*, and *PPP2R2B*, are methylated in gliomas and *SFRP1* methylation predicts shorter survival

**DOI:** 10.1007/s13353-015-0312-7

**Published:** 2015-09-04

**Authors:** Aleksandra Majchrzak-Celińska, Marta Słocińska, Anna-Maria Barciszewska, Stanisław Nowak, Wanda Baer-Dubowska

**Affiliations:** Department of Pharmaceutical Biochemistry, Poznan University of Medical Sciences, Poznań, Poland; Department and Clinic of Neurosurgery and Neurotraumatology, Poznan University of Medical Sciences, Poznań, Poland

**Keywords:** Wnt pathway, *SFRP1*, *SFRP2*, *SOX17*, *PPP2R2B*, DNA methylation

## Abstract

The deregulation of Wnt signaling is observed in various cancers, including gliomas, and might be related to the methylation of the genes encoding antagonists of this signaling pathway. The aim of the study was to assess the methylation status of the promoter regions of six Wnt negative regulators and to determine their prognostic value in clinical samples of gliomas of different grades. The methylation of *SFRP1*, *SFRP2*, *PPP2R2B*, *DKK1*, *SOX17*, and *DACH1* was analyzed in 64 glioma samples using methylation-specific polymerase chain reaction (MSP). The results were analyzed in correlation with clinicopathological data. Promoter methylation in at least one of the analyzed genes was found in 81.3 % of the tumors. All benign tumors [grade I according to the World Health Organization (WHO) classification] lacked the methylation of the studied genes, whereas grade II, III, and IV tumors were, in most cases, methylation-positive. The methylation index correlated with the patient’s age. The most frequently methylated genes were *SFRP1* and *SFRP2* (73.4 % and 46.9 %, respectively), followed by *SOX17* (20.3 %) and *PPP2R2B* (10.9 %); *DKK1* and *DACH1* were basically unmethylated (1.6 %). *SFRP1* methylation negatively correlated with patients’ survival time, and was significantly more frequent in older patients and those with higher grade tumors. Overall, the results of this study indicate that aberrant promoter methylation of Wnt pathway antagonists is common in gliomas, which may be the possible cause of up-regulation of this signaling pathway often observed in these tumors. Moreover, *SFRP1* promoter methylation can be regarded as a potential indicator of glioma patients’ survival.

## Introduction

Gliomas are the most common primary brain tumors in adults, with a wide spectrum of different tumor types. According to the World Health Organization (WHO), they can be divided into low-grade gliomas (WHO grades I and II) and high-grade gliomas (WHO grades III and IV). The most aggressive form of glioma, glioblastoma multiforme (GBM), is basically incurable and the most lethal among solid tumors (Schiefer et al. 2014). In adult patients, it is a rule that low-grade gliomas tend to progress to higher grade malignancies (Ostrom et al. 2014). Unraveling molecular routes leading to glioma development and searching for new diagnostic, prognostic, or predictive biomarkers remains crucial for better diagnosis and more efficient management of these tumors.

**Fig. 1:**
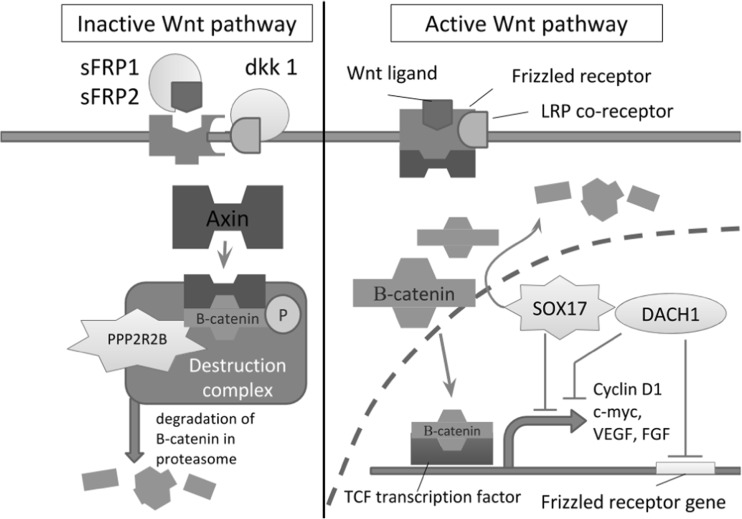
The role of proteins encoded by genes analyzed in this study in the inhibition of the Wnt signaling. The secreted Frizzled-related proteins sFRP1 and sFRP2 are receptors for secreted Wnt proteins, as well as other ligands. The interaction between sFRPs and Wnt proteins prevents the latter from binding the Frizzled receptors (Pannone et al. 2010). The Wnt inhibitor Dkk1 encoded by the *DKK1* gene acts also at the cell membrane level through binding the Frizzled co-receptor LRP, causing its internalization (Zhou et al. 2010). The protein encoded by the *PPP2R2B* gene is a part of the β-catenin degrading complex (Tan et al. 2010). The last two proteins encoded by the *SOX17* and *DACH1* genes act as transcription factors inhibiting the expression of the Wnt pathway target genes. SOX17 also degrades β-catenin independently of the degrading complex (Sinner et al. 2007), while DACH1 inhibits the expression of the Frizzled receptor protein (Wu et al. 2003; Yan et al. 2013)

In recent years, epigenetic biomarkers based on the analysis of the DNA methylation profile have proven to be useful in the detection of and prognostication in various cancer types (Paluszczak and Baer-Dubowska 2006; Majchrzak-Celińska et al. 2013, 2015a, b; Ellinger et al. 2015). In this regard, our previous study showed that DNA methylation of a panel of selected genes can serve as a tool for the prediction of glioma aggressiveness. Moreover, among the individual genes, *RUNX3*, encoding a negative regulator of the Wnt/β-catenin signaling pathway, was found to be the most promising candidate for a new biomarker (Majchrzak-Celińska et al. 2015a). The activation of Wnt/β-catenin signaling promotes a large spectrum of cellular processes, such as proliferation, differentiation, cell adhesion, and migration (Rao and Kühl 2010). In the absence of Wnt ligands, Axin/APC/GSK3β/β-catenin together form a multiprotein destruction complex in which β-catenin is phosphorylated by GSK3β and subsequently degraded by the proteasome. In the nucleus, in the absence of β-catenin, TCF/LEF proteins actively repress target genes through the recruitment of transcriptional co-repressors such as C-terminal-binding protein (CtBP) and Groucho (Gro) (Paul et al. 2013; Zhang et al. 2012). When Wnt ligands bind to Frizzled receptors, the transmembrane Dishevelled protein is activated, which inhibits the activity of GSK3β, thereby blocking the degradation of β-catenin. The accumulation of β-catenin in the cytoplasm leads to its translocation to the nucleus, where it binds to the TCF/LEF family of transcription factors and stimulates the transcription of multiple target genes (Paul et al. 2013; Zhang et al. 2012) (Fig. 1).

The imbalance in the structural and signaling properties of β-catenin often results in deregulated cellular growth related to cancer and metastasis (Kaur et al. 2013; Paluszczak et al. 2014, 2015; Surana et al. 2014). The up-regulation of Wnt signaling was also observed in gliomas and it was suggested that it might be related not only to enhanced cancer cell proliferation, but also to radio- and chemoresistance (Schiefer et al. 2014). Multiple ways of deregulation of the Wnt/β-catenin pathway were proposed and several aberrantly expressed molecules were indicated as potential biomarkers. For instance, increased β-catenin expression has been observed in astrocytic tumors, which correlated with poor prognosis and short survival of GBM patients (Liu et al. 2011; Rossi et al. 2011). Also, the inactivation of key components of the β-catenin degradation complex, such as Axin, was found to be common in brain tumors and, importantly, the levels of Axin correlated negatively with the grade of astrocytoma (Zhang et al. 2009). Recent studies supporting a role for a deregulated Wnt/ β-catenin pathway in malignant glioma also showed that Wnt pathway antagonists such as *WIF1* and a family of secreted Frizzled-related proteins, dickkopf, and naked are epigenetically inactivated as a result of their promoters’ hypermethylation (Lambiv et al. 2011; Götze et al. 2010). However, little is still known about the role of the Wnt pathway in the malignant behavior of human glioma. Moreover, most of the studies on the epigenetic inactivation of Wnt/β-catenin pathway antagonists were performed using cell line models or tested only a small number of genes (Schiefer et al. 2014; Kim et al. 2013).

The aim of the present study was to assess the frequency of the promoter methylation of genes encoding two members of secreted Frizzled-related protein family (*SFRP1*, *SFRP2*), one dickkopf family member (*DKK1*), and three others, namely *PPP2R2B*, *SOX17*, and *DACH1*, all acting as Wnt pathway negative regulators. The genes were selected on the basis of their ability to inhibit different levels of the Wnt pathway (Fig. 1) and their potential role in glioma carcinogenesis. In order to establish the diagnostic or prognostic potential of the studied genes, the correlation with important clinicopathological data such as tumor grade according to WHO classification criteria, patients’ age, gender, and survival time was evaluated.

We found the hypermethylation of *SFRP1* and *SFRP2* gene promoters to be the most frequent. Moreover, correlation of *SFRP1* methylation with tumor grade and patients’ survival may suggest its potential as a prognostic biomarker for glioma patients.

## Materials and methods

### Patients

The study group consisted of 64 patients with glial tumors who were primarily treated surgically at the Department and Clinic of Neurosurgery and Neurotraumatology of Poznan University of Medical Sciences between 2010 and 2013. The histological types of the tumors as well as tumor grades (according to the 2007 WHO classification criteria) were analyzed in the Laboratory of Neuropathology. Twenty-six patients were diagnosed with WHO grade IV glioma, twenty-three with grade III, nine with grade II, and four with grade I tumors. Two patients were not classified according to the WHO grading scale. Women comprised 43.75 % (28/64) and men 56.25 % (36/64) of all patients, and the average patient age was 52 years (median 56 years), ranging from 16 to 83 years. The more detailed characteristics of the studied group is presented in Table 1. Directly after resection, tumor samples were frozen and stored at −80 °C.

**Table 1:** Characteristics of the studied group of patients

A						
Type of tumor			Number of cases		Percentage	
Astrocytic tumors			55		85.94 %	
Oligodendroglial tumors			1		1.56 %	
Oligoastrocytic tumors			2		3.13 %	
Ependymal tumors			1		1.56 %	
Neuronal and mixed neuronal-glial tumors			3		4.69 %	
Unclassified			2		3.13 %	
B						
WHO tumor grade			Number of cases		Percentage	
I*			4		6.25 %	
II**			9		14.06 %	
III			23		35.94 %	
IV			26		40.63 %	
Unclassified			2		3.13 %	
*Including one case classified as I/II						
**Including one case classified as II/III						
C						
Gender			Number of cases		Percentage	
Women			28		43.75 %	
Men			36		56.25 %	
D						
Gender	Age, range (years)		Age, average (years)		Age, median (years)	
Women	19–77		55		56	
Men	16–83		50		56	
E						
The number of patients with Karnofsky Performance Status ≥70			48			
The number of patients with Karnofsky Performance Status <70			16			

The follow-up observation in most cases covered at least 2 years following tumor resection and the information about patients’ overall survival time was available for more than half of the patients (34/64). All the patients gave informed consent for the analyses to be undertaken and the study protocol was approved by the Clinical Research Ethics Committee (approval no. 505/12).

### Isolation of DNA from tumor tissue

DNA from tumor samples was isolated using the GeneMATRIX Tissue DNA Purification Kit (EURx, Gdańsk, Poland), following the manufacturer’s instructions. DNA concentration and purity were measured using a NanoDrop Spectrophotometer and then the DNA was stored at −20 °C for further analysis.

### Methylation-specific polymerase chain reaction (MSP)

**Fig. 2:**
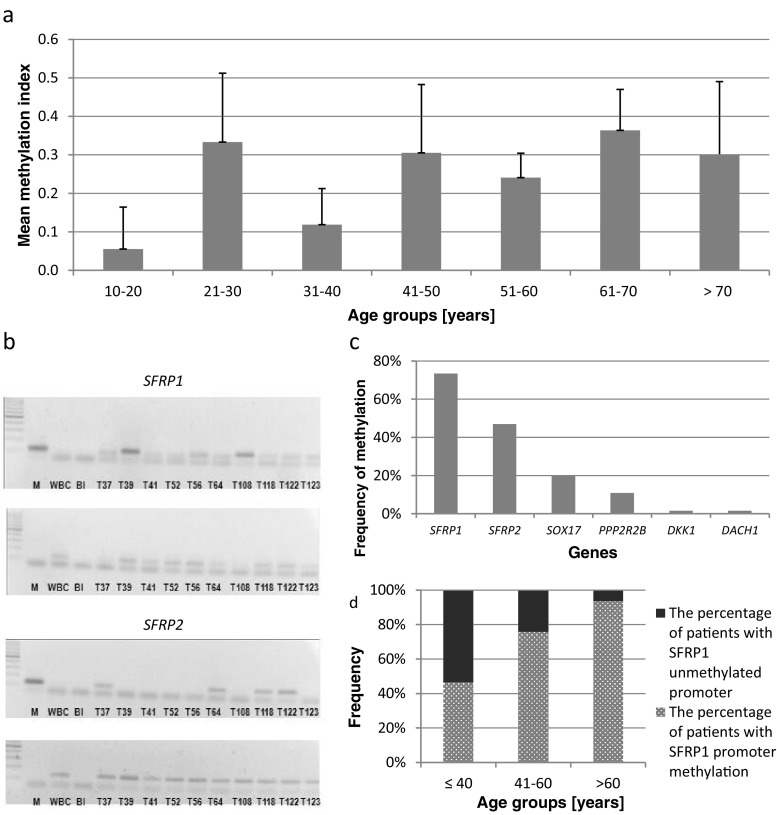
The visualization of the most important results and representative electropherograms presenting the method of the analysis. **a** Mean methylation index in different age groups with 95 % confidence intervals. **b** Representative methylation-specific polymerase chain reaction (MSP) electropherograms of *SFRP1* and *SFRP2* promoter methylation analysis. For each gene, the upper part represents the reaction with primers specific for methylated sequence, whereas the lower part represents the reaction with primers binding to unmethylated sequence. *M* completely methylated human genomic DNA used as positive control; *WBC* white blood cells used as negative control; *Bl* blank control; *T* tumor. **c** The frequency of Wnt antagonists’ promoter methylation. **d** The relationship between promoter methylation of *SFRP1* and patients’ age

Bisulfite modification of 500 ng DNA was performed using the EZ DNA Methylation Kit (Zymo Research, Irvine, USA). *SFRP1*, *SFRP2*, *PPP2R2B*, *DKK1*, *SOX17*, and *DACH1* promoter methylation was assessed with methylation-specific polymerase chain reaction (MSP). Primers were obtained from Oligo.pl (Warsaw, Poland). Primer sequences along with their corresponding annealing temperature are presented in Table 2. All MSP reactions were performed in a MyCycler Thermal Cycler with Gradient (Bio-Rad, Hercules, USA) or a T100 Thermal Cycler with Gradient (Bio-Rad, Hercules, USA), using HOT FIREPol DNA Polymerase (Solis BioDyne, Tartu, Estonia). The reaction protocol was as follows: polymerase activation at 95 °C for 15 min, 40 cycles of 95 °C for 30 s, annealing at the appropriate temperature for 30 s and 72 °C for 30 s, then final elongation at 72 °C for 5 min and hold at 4 °C. MSP products were separated by electrophoresis on 2 % agarose gels and visualized under UV light illumination after staining with SimplySafe (EURx, Gdańsk, Poland). Representative electropherograms of *SFRP1* and *SFRP2* MSP reactions are presented in Fig. 2b. Completely methylated DNA (CpG Methylated HeLa Genomic DNA, New England Biolabs, Ipswich, USA), DNA extracted from white blood cells of a healthy blood donor (WBC), and water served as positive, negative, and blank controls in MSP reactions, respectively. Non-cancerous brain tissue from a patient with brain hematoma and Human Astrocyte Genomic DNA (ScienCell Research Laboratories, Carlsbad, USA) served as additional controls for methylation status in non-cancerous brain tissue.

**Table 2:** Primer sequences with their annealing temperature used for methylation-specific polymerase chain reaction (MSP) analysis

Gene symbol	Primer sequence [5′–3′]		Primer annealing temperature (°C)
*SFRP1*	MF	TGTAGTTTTCGGAGTTAGTGTCGCGC	60 °C
	MR	CCTACGATCGAAAACGACGCGAACG	
	UF	GTTTTGTAGTTTTTGGAGTTAGTGTTGTGT	
	UR	CTCAACCTACAATCAAAAACAACACAAACA	
*SFRP2*	MF	GGGTCGGAGTTTTTCGGAGTTGCGC	60 °C
	MR	CCGCTCTCTTCGCTAAATACGACTCG	
	UF	TTTTGGGTTGGAGTTTTTTGGAGTTGTGT	
	UR	AACCCACTCTCTTCACTAAATACAACTCA	
*PPP2R2B*	MF	AGTAGTAGTTGCGAGTGCGC	61 °C
	MR	GAACAACCGCGACAAAATAAT	
	UF	AGTAGTAGTAGTTGTGAGTGTGT	
	UR	AAACAACCACAACAAAATAATACC	
*DKK1*	MF	GTCGGAATGTTTCGGTTCGC	60 °C
	MR	CTAAATCCCCACGAAACCGTACCG	
	UF	GGGGTTGGAATGTTTTGGGTTTGT	
	UR	ACCTAAATCCCCACAAAACCATACCA	
*SOX17*	MF	GGGGCGTTCGTAGTGTTATTAGGTC	60 °C
	MR	AAACACTAAAATACCCCGAAAACTACG	
	UF	TTAGGGGTGTTTGTAGTGTTATTAGGTT	
	UR	TAAAACACTAAAATACCCCAAAAACTACA	
*DACH1*	MF	GGAAAAAATTATTAGTTTTCGCGGAC	60 °C
	MR	AAACCGAAAACACAAAAATAACGATCG	
	UF	TTTGGAAAAAATTATTAGTTTTTGTGGAT	
	UR	AAAAAACCAAAAACACAAAAATAACAATCA

### Statistical analysis

The correlation between clinicopathological features and gene methylation was assessed using CSS Statistica, version 10. The Chi-square test was used to determine the correlations between detected methylation and patients’ age, gender, and tumor WHO grade, and Spearman’s rank correlation coefficient was applied to measure the dependence between the methylation index (MI) and patients’ age or tumor WHO grade, while the Mann–Whitney *U*-test verified if promoter methylation correlates with patients’ survival time.

## Results

Methylation in at least one of the six analyzed genes occurred in 81.3 % of the tumors, while 46.9 % of the cases showed methylation in at least two genes. None of the genes were methylated in DNA derived from either normal human astrocytes or non-cancerous brain tissue. This indicates that the observed changes are cancer-specific. All benign, grade I tumors had no methylated genes detected, whereas grade II, III, and IV tumors were, in most cases, methylation-positive. However, the average MI, defined as the number of methylated genes divided by the number of all genes analyzed in the panel (Chen et al. 2011), did not differ significantly between groups of patients with different WHO grades. Neither did it for patients grouped according to their gender. Nevertheless, the MI of individual patients correlated with their age (*p* = 0.02); that is, older patients (>40 years of age) had more methylated loci as compared to younger ones (<40 years of age) (Fig. 2a).

As shown in Fig. 2c, the highest frequency of methylation was detected for *SFRP1* and *SFRP2* (73.4 % and 46.9 %, respectively), followed by *SOX17* (20.3 %) and *PPP2R2B* (10.9 %) genes. *DKK1* and *DACH1* were basically unmethylated (1.6 %). The methylation of individual genes did not correlate with gender. However, *SFRP1* methylation was found to be significantly more frequent in older patients as compared to younger ones (*p* = 0.01). Almost all of the patients (93.75 %) over 60 years of age showed the presence of *SFRP1* promoter methylation, whereas the methylation of this gene was detected less frequently in younger patient groups (41–60 years 75.76 %, <40 years 46.67 %) (Fig. 2d).

**Fig. 3:**
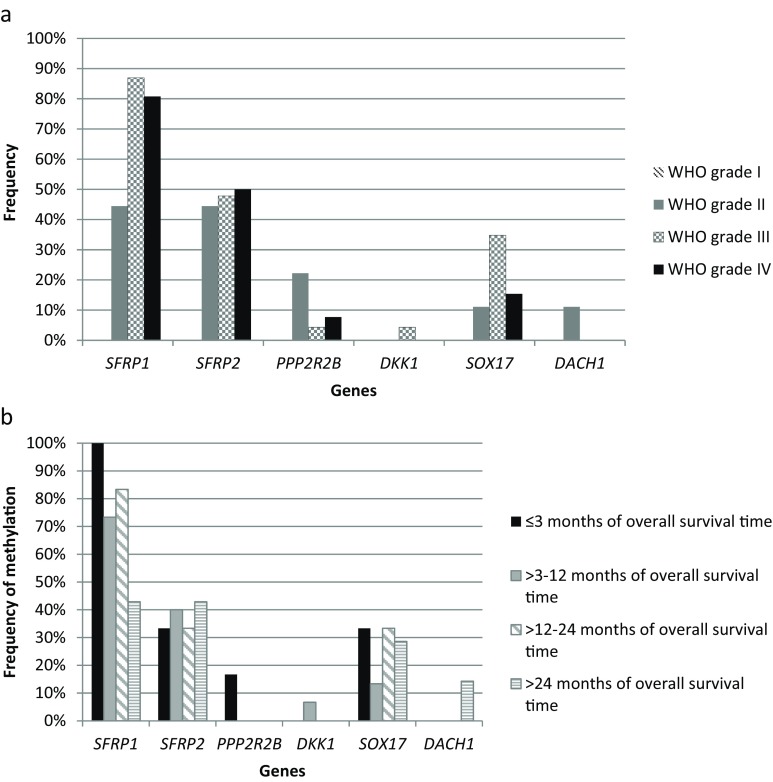
The relationship between the frequency of Wnt antagonists’ methylation with tumor grade and patients’ overall survival time. **a** The relationship between the frequency of Wnt antagonists’ promoter methylation and tumor grade according to the World Health Organization (WHO) classification. **b** The relationship between the frequency of Wnt antagonists’ promoter methylation and patients’ overall survival time

The results were also analyzed in terms of the correlation with the WHO classification grade of the tumor and are presented in Fig. 3a. *SFRP1* methylation was more frequent in patients with higher grade tumors (III and IV). Additionally, *SFRP1* methylation showed a significant negative correlation with patient survival time (Fig. 3b). All patients with the shortest survival (within 1–3 months after tumor resection) had *SFRP1* promoter methylated, whereas the frequency of *SFRP1* methylation in patients who survived longer was lower (73 % and 83 % methylated tumor samples in the “3–12 months” and “12–24 months after tumor resection” groups, respectively). In the long survival group (>24 months), only 43 % of patients showed *SFRP1* promoter methylation. We also observed a trend towards longer survival accompanied by lower average MI, calculated for each group of patients according to their survival time; however, it did not reach statistical significance.

## Discussion

Deregulation of the Wnt pathway, as shown in several reports, is associated with malignant tumor behavior (Fodde and Brabletz 2007; Delic et al. 2014; Zhang et al. 2012). In gliomas, modulation of Wnt signaling has been shown to affect cell growth and motility (Lu et al. 2008; Caricasole et al. 2005; Gong and Huang 2012). Importantly, epigenetic silencing of genes encoding proteins known to act as antagonists of the Wnt network was proposed as one of the mechanisms of up-regulation of the Wnt pathway in malignant gliomas (Foltz et al. 2010).

In this study, we analyzed the methylation status of six genes which encode proteins acting as Wnt signaling negative regulators. Beside the genes, which were described earlier as possible targets of epigenetic silencing in gliomas (*SFRP1*, *SFRP2*, *DKK1*), the methylation of *PPP2R2B*, *SOX17*, and *DACH1* was also assessed. The results showed the methylation of all the analyzed genes, but to a different extent. However, the aberrant methylation was observed only in WHO grade II, III, and IV gliomas; none of the benign grade I tumors showed methylation of any of the studied genes. The high prevalence of methylation of the promoter regions of genes encoding Wnt pathway antagonists in higher grade gliomas indicates that this epigenetic mechanism contributes to the malignant behavior of these CNS tumors. Direct correlation between tumor aggressiveness and promoter methylation was found for one of the analyzed Wnt pathway inhibitors, namely *SFRP1*. Although the MI values did not allow for the differentiation of advanced tumor grades, the MI of an individual patient correlated with his/her age. Older patients had more methylated loci detected than younger ones. Higher MI observed in older patients may result from the alterations in the activity of enzymes involved in epigenetic modifications, which is a phenomenon typically associated with aging (Cencioni et al. 2013) or might also be the result of the accumulation of gene methylation changes related to cancer progression.

Among the individual genes, the most frequent methylation was observed in the case of *SFRP1*. This gene encoding secreted Frizzled-related protein 1 was found to be methylated in almost three quarters of the analyzed glioma samples (73.4 %), and its methylation correlated significantly with patients’ age. As mentioned previously, *SFRP1* methylation was found to be related to tumor aggressiveness and, more importantly, negatively correlated with patients’ survival time. While hypermethylation of this gene in malignant astrocytic gliomas has already been described (Götze et al. 2010), the above-mentioned correlations are novel findings in relation to glioma and may suggest a potential prognostic value of *SFRP1* methylation in this subtype of cancer. This suggestion is further supported by recent data on *SFRP1* mRNA levels in glioma patients presented in the REMBRANT (Repository of Molecular Brain Neoplasia Data) and TCGA (The Cancer Genome Atlas) databases. These data show a significantly shorter overall survival of glioma patients with low *SFRP1* expression compared with patients with high *SFRP1* transcript levels (Delic et al. 2014). Our results indirectly indicate that the hypermethylation of *SFRP1* promoter sequence may be the main mechanism for the decrease in the level of its expression. Taking into consideration the possible clinical application of these findings, reversal of aberrant gene methylation through the use of epigenome modifying agents opens the possibility to restore the expression of *SFRP1* or other SFRP family members and, therefore, antagonize deleterious Wnt signaling (Surana et al. 2014). This possibility has already been tested in glioma cell lines and was proven to be successful (Schiefer et al. 2014; Surana et al. 2014).

Frequent methylation (55 %) of *SFRP2* was also observed, but its relationship to glioma aggressiveness was not as straightforward as in the case of *SFRP1*. The methylation of *SFRP2* did not correlate with either tumor grade or survival time. Nevertheless, since SFRP1 and SFRP2 proteins are structurally similar to the extracellular Wnt binding domain of the Frizzled receptors, they can antagonize Wnt signaling by sequestering Wnt ligands through the Frizzled cysteine-rich domain (CRD) or by forming inactive complexes with the Frizzled receptors (Hendaoui et al. 2012). Thus, it is possible that the silencing of those two inhibitors is sufficient for Wnt pathway activation in glioma patients. This might explain the lower methylation frequency of the other genes analyzed in our current study. Despite the apparent similar roles of SFRP1 and SFRP2 proteins, the significance of *SFRP2* silencing in human cancers is generally less clear. It has been reported that, besides acting as a Wnt antagonist, SFRP2 can also play a role as an enhancer of Wnt/β-catenin signaling (von Marschall and Fisher 2010).

The SRY-box containing gene 17 (*SOX17*) was methylated in around one fifth (20.31 %) of patients. Even though this may seem low compared to other tumor types [for instance, 82 % methylated samples of human hepatocellular carcinoma (Jia et al. 2010) or 93 %, 100 %, and 94 % methylated samples of nonpolypoid adenomas, polypoid adenomas, and colorectal carcinomas, respectively (Voorham et al. 2013)], the relevance of this epigenetic change can possibly be of great importance, at least in a subset of glioma patients. The studies performed on rat and mouse models provided evidence that Sox17 regulates the Wnt/β-catenin signaling pathway in oligodendrocyte progenitor cells (Chew et al. 2011; Sohn et al. 2006). *Sox17* knockdown increased the levels of cyclin D1, Axin2, and activated β-catenin (Chew et al. 2011). The precise role of *SOX17* in human glioma tumor cells, or specifically oligodendroglioma tumor cells, has not yet been evaluated. However, in our study, *SOX17* was found methylated in anaplastic oligodendroglioma samples, so we speculate that epigenetic silencing of this gene may function as a factor up-regulating the Wnt pathway or deregulating the cell cycle in these tumors.

As far as *PPP2R2B* is concerned, recent studies show that its promoter is methylated in colorectal cancer (Tan et al. 2010), ductal carcinoma in situ, and early invasive breast cancer (Muggerud et al. 2010), as well as laryngeal squamous cell carcinoma (Paluszczak et al. 2014). Here, we report that, also, roughly one tenth of glioma tumor samples (10.9 %) is characterized by this epigenetic change. We also show that *DACH1* and *DKK1* methylation is an infrequent event in glioma. This observation is worth noting, considering the fact that, to date, no or only few studies have addressed this issue. Mechanisms other than epigenetic were proposed for *DACH1* silencing, e.g., homozygous deletion of the chromosomal region containing *DACH1* gene sequence (13q21) (Watanabe et al. 2011). Interestingly, the induction of the expression of *DACH1* decreased cell proliferation in a series of glioma cell lines, whereas loss of *DACH1* increased the number of tumor-initiating cells through transcriptional activation of bFGF (Watanabe et al. 2011). In contrast to *DACH1*, epigenetic changes were reported to play a role in reducing *DKK1* expression in gliomas. In this regard, Foltz et al. (2010) showed that, among three Wnt antagonist genes, *DKK1*, *SFRP1*, and *WIF-1*, only *DKK1* expression was restored by treatment with DNA demethylating agent 5-azacytidine in T98 glioblastoma cells, suggesting the presence of promoter hypermethylation. However, in contrast to the data presented by other authors (Götze et al. 2010; Foltz et al. 2010), our study showed negligible frequency of *DKK1* methylation. One reason for this discrepancy might be the origin of glioma samples, as Götze et al. (2010) have found *DKK1* hypermethylation in 50 % of secondary but not primary glioblastoma.

The overall results of our current study, together with the hypermethylation of *RUNX3* observed in our earlier investigation, indicate that epigenetic silencing of Wnt antagonists may constitute a crucial mechanism of abnormal up-regulation of this signaling pathway which is often observed in gliomas. Moreover, *SFRP1* methylation can be regarded as a potential indicator of glioma patient survival.

## References

[CR1] Caricasole A, Bakker A, Copani A, Nicoletti F, Gaviraghi G, Terstappen GC (2005). Two sides of the same coin: Wnt signaling in neurodegeneration and neuro-oncology. Biosci Rep.

[CR2] Cencioni C, Spallotta F, Martelli F, Valente S, Mai A, Zeiher AM, Gaetano C (2013). Oxidative stress and epigenetic regulation in ageing and age-related diseases. Int J Mol Sci.

[CR3] Chen PC, Tsai MH, Yip SK, Jou YC, Ng CF, Chen Y, Wang X, Huang W, Tung CL, Chen GC, Huang MM, Tong JH, Song EJ, Chang DC, Hsu CD, To KF, Shen CH, Chan MW (2011). Distinct DNA methylation epigenotypes in bladder cancer from different Chinese sub-populations and its implication in cancer detection using voided urine. BMC Med Genomics.

[CR4] Chew LJ, Shen W, Ming X, Senatorov VV Jr, Chen HL, Cheng Y, Hong E, Knoblach S, Gallo V (2011) SRY-box containing gene 17 regulates the Wnt/β-catenin signaling pathway in oligodendrocyte progenitor cells. J Neurosci 39:13921–13935 10.1523/JNEUROSCI.3343-11.2011 PMC3227525 21957254

[CR5] Delic S, Lottmann N, Stelzl A, Liesenberg F, Wolter M, Götze S, Zapatka M, Shiio Y, Sabel MC, Felsberg J, Reifenberger G, Riemenschneider MJ (2014). MiR-328 promotes glioma cell invasion via SFRP1-dependent Wnt-signaling activation. Neuro Oncol.

[CR6] Ellinger J, Müller SC, Dietrich D (2015). Epigenetic biomarkers in the blood of patients with urological malignancies. Expert Rev Mol Diagn.

[CR7] Fodde R, Brabletz T (2007). Wnt/beta-catenin signaling in cancer stemness and malignant behavior. Curr Opin Cell Biol.

[CR8] Foltz G, Yoon JG, Lee H, Ma L, Tian Q, Hood L, Madan A (2010). Epigenetic regulation of wnt pathway antagonists in human glioblastoma multiforme. Genes Cancer.

[CR9] Gong A, Huang S (2012). FoxM1 and Wnt/β-catenin signaling in glioma stem cells. Cancer Res.

[CR10] Götze S, Wolter M, Reifenberger G, Müller O, Sievers S (2010). Frequent promoter hypermethylation of Wnt pathway inhibitor genes in malignant astrocytic gliomas. Int J Cancer.

[CR11] Hendaoui I, Lavergne E, Lee HS, Hong SH, Kim HZ, Parent C, Heuzé-Vourc’h N, Clément B, Musso O (2012). Inhibition of Wnt/β-catenin signaling by a soluble collagen-derived frizzled domain interacting with Wnt3a and the receptors frizzled 1 and 8. PLoS One.

[CR12] Jia Y, Yang Y, Liu S, Herman JG, Lu F, Guo M (2010). SOX17 antagonizes WNT/β-catenin signaling pathway in hepatocellular carcinoma. Epigenetics.

[CR13] Kaur N, Chettiar S, Rathod S, Rath P, Muzumdar D, Shaikh ML, Shiras A (2013). Wnt3a mediated activation of Wnt/β-catenin signaling promotes tumor progression in glioblastoma. Mol Cell Neurosci.

[CR14] Kim SA, Kwak J, Nam HY, Chun SM, Lee BW, Lee HJ, Khang SK, Kim SW (2013). Promoter methylation of WNT inhibitory factor-1 and expression pattern of WNT/β-catenin pathway in human astrocytoma: pathologic and prognostic correlations. Mod Pathol.

[CR15] Lambiv WL, Vassallo I, Delorenzi M, Shay T, Diserens AC, Misra A, Feuerstein B, Murat A, Migliavacca E, Hamou MF, Sciuscio D, Burger R, Domany E, Stupp R, Hegi ME (2011). The Wnt inhibitory factor 1 (WIF1) is targeted in glioblastoma and has a tumor suppressing function potentially by induction of senescence. Neuro Oncol.

[CR16] Liu X, Wang L, Zhao S, Ji X, Luo Y, Ling F (2011). β-Catenin overexpression in malignant glioma and its role in proliferation and apoptosis in glioblastma cells. Med Oncol.

[CR17] Lu J, Zhang F, Zhao D, Hong L, Min J, Zhang L, Li F, Yan Y, Li H, Ma Y, Li Q (2008). ATRA-inhibited proliferation in glioma cells is associated with subcellular redistribution of beta-catenin via up-regulation of Axin. J Neurooncol.

[CR18] Majchrzak-Celińska A, Paluszczak J, Kleszcz R, Magiera M, Barciszewska AM, Nowak S, Baer-Dubowska W (2013). Detection of MGMT, RASSF1A, p15INK4B, and p14ARF promoter methylation in circulating tumor-derived DNA of central nervous system cancer patients. J Appl Genet.

[CR19] Majchrzak-Celińska A, Paluszczak J, Szalata M, Barciszewska AM, Nowak S, Kleszcz R, Sherba A, Baer-Dubowska W (2015a) The methylation of a panel of genes differentiates low-grade from high-grade gliomas. Tumour Biol 36:3831–3841. doi:10.1007/s13277-014-3025-3 10.1007/s13277-014-3025-3 25563195

[CR20] Majchrzak-Celińska A, Paluszczak J, Szalata M, Barciszewska AM, Nowak S, Baer-Dubowska W (2015). DNA methylation analysis of benign and atypical meningiomas: correlation between RUNX3 methylation and WHO grade. J Cancer Res Clin Oncol.

[CR21] Muggerud AA, Rønneberg JA, Wärnberg F, Botling J, Busato F, Jovanovic J, Solvang H, Bukholm I, Børresen-Dale AL, Kristensen VN, Sørlie T, Tost J (2010). Frequent aberrant DNA methylation of ABCB1, FOXC1, PPP2R2B and PTEN in ductal carcinoma in situ and early invasive breast cancer. Breast Cancer Res.

[CR22] Ostrom QT, Bauchet L, Davis FG, Deltour I, Fisher JL, Langer CE, Pekmezci M, Schwartzbaum JA, Turner MC, Walsh KM, Wrensch MR, Barnholtz-Sloan JS (2014). The epidemiology of glioma in adults: a “state of the science” review. Neuro Oncol.

[CR23] Paluszczak J, Baer-Dubowska W (2006). Epigenetic diagnostics of cancer—the application of DNA methylation markers. J Appl Genet.

[CR24] Paluszczak J, Hemmerling D, Kostrzewska-Poczekaj M, Jarmuż-Szymczak M, Grenman R, Wierzbicka M, Baer-Dubowska W (2014). Frequent hypermethylation of WNT pathway genes in laryngeal squamous cell carcinomas. J Oral Pathol Med.

[CR25] Paluszczak J, Sarbak J, Kostrzewska-Poczekaj M, Kiwerska K, Jarmuż-Szymczak M, Grenman R, Mielcarek-Kuchta D, Baer-Dubowska W (2015). The negative regulators of Wnt pathway-DACH1, DKK1, and WIF1 are methylated in oral and oropharyngeal cancer and WIF1 methylation predicts shorter survival. Tumour Biol.

[CR26] Pannone G, Bufo P, Santoro A, Franco R, Aquino G, Longo F, Botti G, Serpico R, Cafarelli B, Abbruzzese A, Caraglia M, Papagerakis S, Lo Muzio L (2010). WNT pathway in oral cancer: epigenetic inactivation of WNT-inhibitors. Oncol Rep.

[CR27] Paul I, Bhattacharya S, Chatterjee A, Ghosh MK (2013). Current understanding on EGFR and Wnt/β-catenin signaling in glioma and their possible crosstalk. Genes Cancer.

[CR28] Rao TP, Kühl M (2010). An updated overview on Wnt signaling pathways: a prelude for more. Circ Res.

[CR29] Rossi M, Magnoni L, Miracco C, Mori E, Tosi P, Pirtoli L, Tini P, Oliveri G, Cosci E, Bakker A (2011). β-Catenin and Gli1 are prognostic markers in glioblastoma. Cancer Biol Ther.

[CR30] Schiefer L, Visweswaran M, Perumal V, Arfuso F, Groth D, Newsholme P, Warrier S, Dharmarajan A (2014). Epigenetic regulation of the secreted frizzled-related protein family in human glioblastoma multiforme. Cancer Gene Ther.

[CR31] Sinner D, Kordich JJ, Spence JR, Opoka R, Rankin S, Lin SC, Jonatan D, Zorn AM, Wells JM (2007). Sox17 and Sox4 differentially regulate beta-catenin/T-cell factor activity and proliferation of colon carcinoma cells. Mol Cell Biol.

[CR32] Sohn J, Natale J, Chew LJ, Belachew S, Cheng Y, Aguirre A, Lytle J, Nait-Oumesmar B, Kerninon C, Kanai-Azuma M, Kanai Y, Gallo V (2006). Identification of Sox17 as a transcription factor that regulates oligodendrocyte development. J Neurosci.

[CR33] Surana R, Sikka S, Cai W, Shin EM, Warrier SR, Tan HJ, Arfuso F, Fox SA, Dharmarajan AM, Kumar AP (2014). Secreted frizzled related proteins: Implications in cancers. Biochim Biophys Acta.

[CR34] Tan J, Lee PL, Li Z, Jiang X, Lim YC, Hooi SC, Yu Q (2010). B55β-associated PP2A complex controls PDK1-directed myc signaling and modulates rapamycin sensitivity in colorectal cancer. Cancer Cell.

[CR35] von Marschall Z, Fisher LW (2010). Secreted Frizzled-related protein-2 (sFRP2) augments canonical Wnt3a-induced signaling. Biochem Biophys Res Commun.

[CR36] Voorham QJ, Janssen J, Tijssen M, Snellenberg S, Mongera S, van Grieken NC, Grabsch H, Kliment M, Rembacken BJ, Mulder CJ, van Engeland M, Meijer GA, Steenbergen RD, Carvalho B (2013). Promoter methylation of Wnt-antagonists in polypoid and nonpolypoid colorectal adenomas. BMC Cancer.

[CR37] Watanabe A, Ogiwara H, Ehata S, Mukasa A, Ishikawa S, Maeda D, Ueki K, Ino Y, Todo T, Yamada Y, Fukayama M, Saito N, Miyazono K, Aburatani H (2011). Homozygously deleted gene DACH1 regulates tumor-initiating activity of glioma cells. Proc Natl Acad Sci U S A.

[CR38] Wu K, Yang Y, Wang C, Davoli MA, D’Amico M, Li A, Cveklova K, Kozmik Z, Lisanti MP, Russell RG, Cvekl A, Pestell RG (2003). DACH1 inhibits transforming growth factor-beta signaling through binding Smad4. J Biol Chem.

[CR39] Yan W, Wu K, Herman JG, Brock MV, Fuks F, Yang L, Zhu H, Li Y, Yang Y, Guo M (2013). Epigenetic regulation of DACH1, a novel Wnt signaling component in colorectal cancer. Epigenetics.

[CR40] Zhang LY, Ye J, Zhang F, Li FF, Li H, Gu Y, Liu F, Chen GS, Li Q (2009). Axin induces cell death and reduces cell proliferation in astrocytoma by activating the p53 pathway. Int J Oncol.

[CR41] Zhang K, Zhang J, Han L, Pu P, Kang C (2012). Wnt/beta-catenin signaling in glioma. J Neuroimmune Pharmacol.

[CR42] Zhou Y, Liu F, Xu Q, Wang X (2010). Analysis of the expression profile of Dickkopf-1 gene in human glioma and the association with tumor malignancy. J Exp Clin Cancer Res.

